# The Metallophore Staphylopine Enables *Staphylococcus aureus* To Compete with the Host for Zinc and Overcome Nutritional Immunity

**DOI:** 10.1128/mBio.01281-17

**Published:** 2017-10-31

**Authors:** Kyle P. Grim, Brian San Francisco, Jana N. Radin, Erin B. Brazel, Jessica L. Kelliher, Paola K. Párraga Solórzano, Philip C. Kim, Christopher A. McDevitt, Thomas E. Kehl-Fie

**Affiliations:** a Department of Microbiology, University of Illinois Urbana-Champaign, Urbana, Illinois, USA; b Institute for Genomic Biology, University of Illinois Urbana-Champaign, Urbana, Illinois, USA; c Research Centre for Infectious Diseases, School of Biological Sciences, University of Adelaide, Adelaide, South Australia, Australia; d Departamento de Ciencias de la Vida, Universidad de las Fuerzas Armadas ESPE, Sangolquí, Ecuador; New York University School of Medicine

**Keywords:** CntABCDF, *Staphylococcus aureus*, calprotectin, nutritional immunity, siderophores, staphylopine, zinc

## Abstract

During infection, the host sequesters essential nutrients, such as zinc, to combat invading microbes. Despite the ability of the immune effector protein calprotectin to bind zinc with subpicomolar affinity, *Staphylococcus aureus* is able to successfully compete with the host for zinc. However, the zinc importers expressed by *S. aureus* remain unknown. Our investigations have revealed that *S. aureus* possesses two importers, AdcABC and CntABCDF, which are induced in response to zinc limitation. While AdcABC is similar to known zinc importers in other bacteria, CntABCDF has not previously been associated with zinc acquisition. Concurrent loss of the two systems severely impairs the ability of *S. aureus* to obtain zinc and grow in zinc-limited environments. Further investigations revealed that the Cnt system is responsible for the ability of *S. aureus* to compete with calprotectin for zinc in culture and contributes to acquisition of zinc during infection. The *cnt* locus also enables *S. aureus* to produce the broad-spectrum metallophore staphylopine. Similarly to the Cnt transporter, loss of staphylopine severely impairs the ability of *S. aureus* to resist host-imposed zinc starvation, both in culture and during infection. Further investigations revealed that together staphylopine and the Cnt importer function analogously to siderophore-based iron acquisition systems in order to facilitate zinc acquisition by *S. aureus*. Analogous systems are found in a broad range of Gram-positive and Gram-negative bacterial pathogens, suggesting that this new type of zinc importer broadly contributes to the ability of bacteria to cause infection.

## IMPORTANCE

A critical host defense against infection is the restriction of zinc availability. Despite the subpicomolar affinity of the immune effector calprotectin for zinc, *Staphylococcus aureus* can successfully compete for this essential metal. Here, we describe two zinc importers, AdcABC and CntABCDF, possessed by *S. aureus*, the latter of which has not previously been associated with zinc acquisition. The ability of *S. aureus* to compete with the host for zinc is dependent on CntABCDF and the metallophore staphylopine, both in culture and during infection. These results expand the mechanisms utilized by bacteria to obtain zinc, beyond Adc-like systems, and demonstrate that pathogens utilize strategies similar to siderophore-based iron acquisition to obtain other essential metals during infection. The staphylopine synthesis machinery is present in a diverse collection of bacteria, suggesting that this new family of zinc importers broadly contributes to the ability of numerous pathogens to cause infection.

## INTRODUCTION

Antibiotic resistance is a serious and growing problem, with both the Centers for Disease Control and Prevention and the World Health Organization calling for the development of new approaches to treat bacterial infections (1, 2). One pathogen of substantial concern is *Staphylococcus aureus*, which colonizes one-third of the world’s population and is a leading cause of antibiotic-resistant infections (3, 4). A promising approach to combating infections is disrupting the ability of bacteria to overcome the host immune response.

During infection, pathogens must overcome host-mediated restriction of essential nutrients, including transition metal ions. This defense, known as nutritional immunity, exploits the crucial roles of metal ions in cellular chemistry. Proteomic analyses suggest that ~30% of all proteins utilize a metal cofactor, emphasizing the scope of potential impact mediated by this defensive mechanism (5, 6). Although originally associated only with iron (Fe) restriction, the breadth of the nutrient withholding response is now known to also include manganese (Mn) and zinc (Zn) (7–11). The power of this defense is demonstrated by the staphylococcal abscess, which is rendered virtually devoid of Mn and Zn (8, 12). This restriction starves invaders of these essential metals, thereby inactivating metal-dependent processes, reducing bacterial growth, and rendering them more sensitive to other aspects of the immune response (8, 10, 13, 14).

A critical component of the Mn and Zn withholding response is the immune effector calprotectin (CP) (8–10). This Mn and Zn binding protein comprises 40 to 60% of the protein in the neutrophil cytoplasm and can be found at sites of infection at concentrations exceeding 1 mg/ml (15, 16). Loss of CP compromises the host metal withholding response and increases susceptibility to infection by *S. aureus* and other Gram-positive, Gram-negative, and fungal pathogens, including *Acinetobacter baumannii*, *Klebsiella pneumoniae*, and *Candida albicans*. In culture, CP-imposed metal starvation is antimicrobial toward these and other pathogens, including *Enterococcus faecalis*, *Pseudomonas aeruginosa*, *Shigella flexneri*, *Salmonella enterica* serovar Typhimurium, and *Aspergillus nidulans* (8, 10, 12, 17–24).

CP is a member of the S100 family of proteins and is a heterodimer comprised of S100A8 and S100A9 (10, 20, 25). This immune effector has two transition metal ion binding sites, a Mn/Zn site and a Zn site (10). The Mn/Zn site is capable of binding either Mn^2+^ or Zn^2+^ with low-nanomolar and picomolar affinities (*K*_*d*_ [dissociation constant] of <1.3 nM for Mn and a *K*_*d*_ between 0.9 pM and 240 pM for Zn) (10, 20, 26–28). Intriguingly, the Mn/Zn site also binds Fe^2+^ (29). However, Fe^3+^ predominates extracellularly during infection, and *in vitro* Fe^2+^ binding does not prevent *S. aureus* or *A. baumannii* from accumulating Fe (12, 13, 17, 30). Thus, the physiological relevance of Fe^2+^ binding by CP, if any, remains to be determined. The Zn site, which binds only Zn^2+^, has subpicomolar affinity for Zn (*K*_*d*_ of <90 fM) (10, 20, 26–28).

Surprisingly, despite the high affinity of CP for Zn, CP variants lacking either the Mn/Zn or Zn site revealed that sequestration of only Zn is insufficient for maximal antimicrobial activity (12, 20). This finding is remarkable as ~6% of *Escherichia coli* proteins are predicted to require Zn as a cofactor, with a similar percentage expected in *S. aureus* and other bacteria (31). The observation that CP concentrations that abrogate Mn acquisition do not prevent Zn accumulation by *S. aureus* explains why Zn restriction is not sufficient for maximal antimicrobial activity (13). It also indicates that despite the high affinity of CP for Zn, *S. aureus* can successfully compete with the host for this metal.

High-affinity metal importers are the best-characterized mechanisms used by pathogens to overcome nutritional immunity (9, 11). These uptake pathways typically occur as those that bind a metal directly, a metal chelate, or a metal-containing host protein (9, 32, 33). In the context of infection, examples of the latter two strategies, which include siderophores and heme/hemoglobin transporters, have been largely restricted to the acquisition of Fe (32, 34). The most widely distributed bacterial Zn importers are the ATP-binding cassette (ABC) permeases of the ZnuABC/AdcABC family. Numerous studies have shown the crucial role of this family in bacterial Zn acquisition and infection, while specific studies have also revealed the contribution of this family to the ability of *A. baumannii*, *S*. Typhimurium, and *P. aeruginosa* to resist CP-imposed Zn limitation (17, 23, 24, 35). The ability of *S. aureus* to compete with CP for Zn is presumably mediated by the expression of high-affinity Zn importers. However, the identity of the Zn importers expressed by *S. aureus* remains unknown.

Given the ability of *S. aureus* to compete with the host for Zn, we sought to elucidate the identity of the staphylococcal Zn importers. We show that *S. aureus* obtains Zn using an AdcABC permease and an additional importer, CntABCDF, which has not previously been associated with Zn uptake. Analysis of CntABCDF, a member of the Opp/NikA family of ABC transporters, shows that it functions in conjunction with the metallophore staphylopine (StP). Further analyses of this system showed that while StP can bind a broad range of first-row transition metals, *S. aureus* employs it as a zincophore. Analysis of the respective roles of the two Zn acquisition systems in overcoming nutritional immunity revealed that the Cnt-StP system, but not AdcABC, is responsible for the ability of *S. aureus* to compete with CP for Zn and serves as the major uptake pathway used by the bacterium to obtain this metal during infection. Further analysis revealed that similar zincophore systems are present in a diverse collection of pathogens. Collectively, these findings significantly expand our understanding of how bacteria obtain this essential nutrient and compete with the host for Zn during infection.

## RESULTS

### *S. aureus* utilizes an uncharacterized import pathway to obtain zinc.

Examination of the staphylococcal genome identified a single putative Zn-associated ABC permease (NWMN_2306, 1458, and 1459). The transporter was designated AdcABC due to its similarity to other Gram-positive Zn-specific ABC permeases. Bioinformatic analysis of AdcA (NWMN_2306), the metal ion-recruiting component of the permease, revealed that it is comprised of two domains. The N-terminal domain (amino acids 1 to 322) belonged to the cluster A-I subgroup of solute binding proteins (SBPs), which have specific roles in Mn, Fe, and Zn recruitment (36). The C-terminal domain (amino acids 323 to 531) showed high similarity to ZinT, a periplasmic metallochaperone present in some Gram-negative bacteria (37). This SBP composition is unique to the Adc permeases of Gram-positive bacteria such as *S. pneumoniae* (38). The ABC transporter AdcBC is comprised of a transmembrane component, AdcB (NWMN_1458), and a nucleotide-binding domain, AdcC (NWMN_1459). Unusually, the gene encoding *S. aureus* AdcA was not clustered with the ABC transporter.

**FIG 1:**
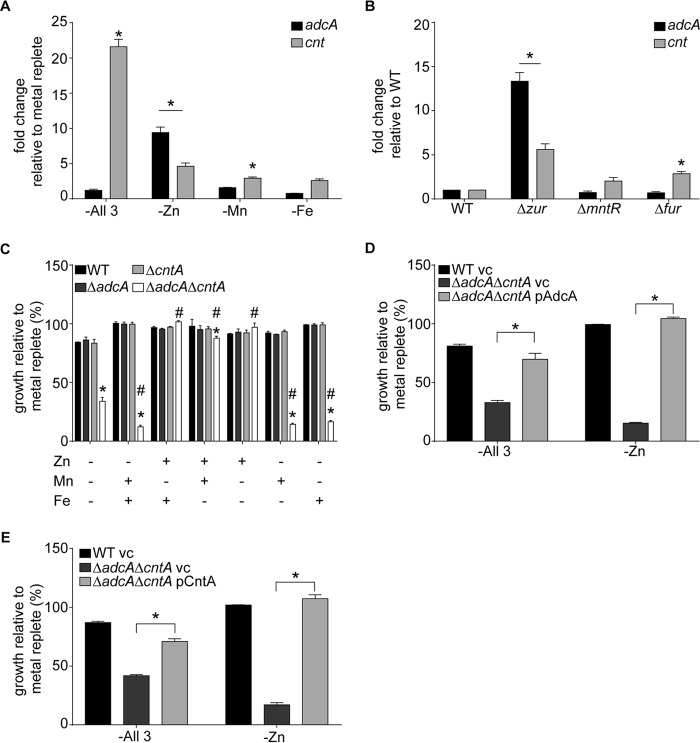
*S. aureus* uses an AdcABC family member and a previously unknown Zn transporter to obtain Zn. (A and B) *S. aureus* containing the P_*adcA*_-YFP and P_*cnt*_-YFP reporter plasmids was grown in NRPMI supplemented with 25 μM ZnSO_4_, 25 μM MnCl_2_, or 1 μM FeSO_4_ or all three metals (metal replete), and expression was assessed by measuring fluorescence. (A) Wild-type *S. aureus* was grown in medium lacking the indicated metal, and expression of *adcA* and the *cnt* loci was assessed. *n* ≥ 3. *, *P* < 0.05 relative to metal-replete medium via two-way analysis of variance with Dunnett’s posttest. (B) Expression of *adcA* and the *cnt* loci was assessed in *Δzur*, *Δfur*, and *ΔmntR* mutants and compared to wild-type (WT) bacteria following growth in metal-replete NRPMI. *n* = 3. *, *P* < 0.05 relative to wild type under the same growth condition via two-way analysis of variance with Dunnett’s posttest. (C) Wild-type *S. aureus* and Δ*adcA*, Δ*cntA*, and Δ*adcA* Δ*cntA* mutants were incubated in NRPMI supplemented with 25 μM ZnSO_4_, 25 μM MnCl_2_, or 25 μM FeSO_4_, and growth was assessed by measuring optical density (OD_600_). *n* = 3. *, *P* < 0.05 compared to wild type via two-way analysis of variance with Dunnett’s posttest. #, *P* < 0.05 compared to Δ*adcA* Δ*cntA* mutant in metal-depleted medium via two-way analysis of variance with Dunnett’s posttest. (D and E) Wild-type *S. aureus* and Δ*adcA*, Δ*cntA*, and Δ*adcA* Δ*cntA* mutants containing the indicated plasmids (vc, vector control) were incubated in NRPMI supplemented with 25 μM MnCl_2_ and 25 μM FeSO_4_ or not, and growth was assessed by measuring optical density (OD_600_). *n* = 3. *, *P* < 0.05 for the indicated comparison via two-way analysis of variance with Tukey’s posttest. Error bars in all panels show standard errors of the means.

Initially, we examined the role of the putative AdcABC permease in Zn acquisition by assessing its transcriptional response to transition metal limitation. Transcription of *adcA* was increased in Zn-depleted medium (Fig. 1A) and in a strain in which the Zn-responsive repressor Zur had been deleted (Fig. 1B) (39). In contrast, no change in *adcA* transcription was observed in Mn- or Fe-depleted medium (Fig. 1A) or in strains that lacked the Mn-responsive regulator MntR or the Fe-responsive regulator Fur (Fig. 1B) (40, 41). Taken together, the *S. aureus* AdcABC system shows structural and transcriptional features consistent with a bacterial Zn importer (17, 42–44). We then investigated the phenotypic impact of metal restriction on an *S. aureus* strain that lacks AdcA (the *ΔadcA* mutant). Limiting Mn and Fe availability had no impact on the growth of the *ΔadcA* mutant (Fig. 1C). Surprisingly, Zn-depleted medium also had no effect on the growth of the *ΔadcA* mutant despite the apparent lack of other Zn import pathways in the staphylococcal genome (Fig. 1C and S1A). Collectively, these findings suggested that in addition to the AdcABC permease, *S. aureus* possesses an import pathway for Zn that is distinct from previously described mechanisms for obtaining this metal.

10.1128/mBio.01281-17.1 FIG S1  Both AdcA and CntA contribute to resistance of *S. aureus* to Zn starvation. (A) Wild-type *S. aureus* and *ΔadcA*, *ΔcntA*, and *ΔadcA ΔcntA* mutants were incubated in NRPMI supplemented with 25 μM MnCl_2_ and 25 μM FeSO_4_ in the presence and absence of 25 μM ZnSO_4_, and growth was assessed by measuring optical density (OD_600_). (B and C) *S. aureus* containing the P_*adcA*_-YFP and P_*cnt*_-YFP reporter plasmids was grown in NRPMI supplemented with various concentrations of CoCl_2_ or NiSO_4_ as indicated, and expression was assessed by measuring fluorescence. (D) Wild-type *S. aureus* and Δ*adcA*, Δ*cntA*, and Δ*adcA* Δ*cntA* mutants were incubated in NRPMI supplemented with 25 μM ZnSO_4_, 25 μM CoCl_2_, or 25 μM NiSO_4_, and growth was assessed by measuring optical density (OD_600_). (E and F) Wild-type *S. aureus* and the *ΔadcA ΔcntA* mutant containing the indicated plasmids (vc, vector control) were incubated in NRPMI supplemented with 25 μM MnCl_2_ and 25 μM FeSO_4_ or not, and growth was assessed by measuring optical density (OD_600_). (G and H) The expression of *adcA* (G) and the *cnt* locus (H) was assessed in a strain lacking AdcA and CntA using the P_*adcA*_-YFP and P_*cnt*_-YFP reporter plasmids, following growth in metal-replete CP assay medium. (A to H) *n* ≥ 3. *, *P* < 0.05 compared to wild type via two-way analysis of variance with Dunnett’s posttest. Error bars show standard errors of the means. Download FIG S1, TIF file, 0.8 MB. Copyright © 2017 Grim et al. 2017 Grim et al. This content is distributed under the terms of the Creative Commons Attribution 4.0 International license.

### A new class of zinc importers facilitates zinc uptake by *S. aureus*.

In Zn-limited medium, in addition to AdcABC, *S. aureus* expresses the Opp/NikA family transporter CntABCDF (45). However, in these studies loss of CntA did not result in a Zn uptake defect. Loss of CntA did reduce the ability of *S. aureus* to transport Co and Ni. These results led to the suggestion that Co and Ni are the physiological substrates of the system (45, 46). Building on our observation that the AdcABC permease is not the sole Zn import pathway, we reevaluated the CntABCDF permease and its potential contribution to Zn import. Consistent with prior results (45), the *cnt* locus was induced in response to Zn limitation (Fig. 1A). Notably, addition of Co and Ni had no impact on *cnt* transcription (Fig. S1B and C). Removal of Fe or Mn from the growth medium also resulted in a modest increase in expression (Fig. 1A). When Zn, Mn, and Fe were omitted from the medium, expression of the system increased by 21-fold, compared to 4.6-fold in medium that lacked only Zn (Fig. 1A). To explore the regulation of this system further, *cnt* transcription in strains lacking Zur, Fur, and MntR was assessed. Loss of Zur and Fur, but not MntR, resulted in increased expression of the *cnt* locus (Fig. 1B). Together, these results show that while the expression of Cnt is responsive to the availability of Fe and Mn, Zn abundance is the dominant factor controlling expression, implying a role in Zn acquisition.

We then evaluated the contribution of the Cnt system to Zn acquisition. To accomplish this goal, we generated a strain lacking CntA (the Δ*cntA* mutant). We observed that, consistent with prior studies (45, 46), there was no discernible growth defect of the Δ*cntA* mutant in Zn-depleted medium (Fig. 1C and S1A). Given the potential for overlapping function with the AdcABC system, we assessed the growth of a strain lacking both potential Zn import pathways (the Δ*adcA* Δ*cntA* mutant). Growth of the double mutant in medium lacking Zn, Fe, and Mn was severely compromised (Fig. 1C and S1C). Supplementation with either Mn, Fe, Ni, or Co failed to reverse the growth defect of the Δ*adcA* Δ*cntA* mutant (Fig. 1C and S1D). Notably, addition of either Mn or Fe further reduced the growth of the Δ*adcA* Δ*cntA* mutant. In contrast, supplementation with Zn restored the growth of the Δ*adcA* Δ*cntA* mutant to wild-type levels. Ectopic expression of either *adcA* or *cntA* also reversed the growth defect of the Δ*adcA* Δ*cntA* mutant in Zn-depleted medium (Fig. 1D and E, and Fig. S1E and F). We also observed that in metal-replete medium, loss of AdcA resulted in increased expression of the *cnt* locus while loss of CntA did not result in increased expression of *adcA* (Fig. S1G and H). This result suggests that AdcA is the first system used by *S. aureus* to obtain Zn, with the Cnt system being induced when the bacterium cannot meet the cellular demand for this metal using the Adc system. Collectively, these results show that both AdcA and CntA contribute to the ability of *S. aureus* to grow in Zn-limited environments.

**FIG 2:**
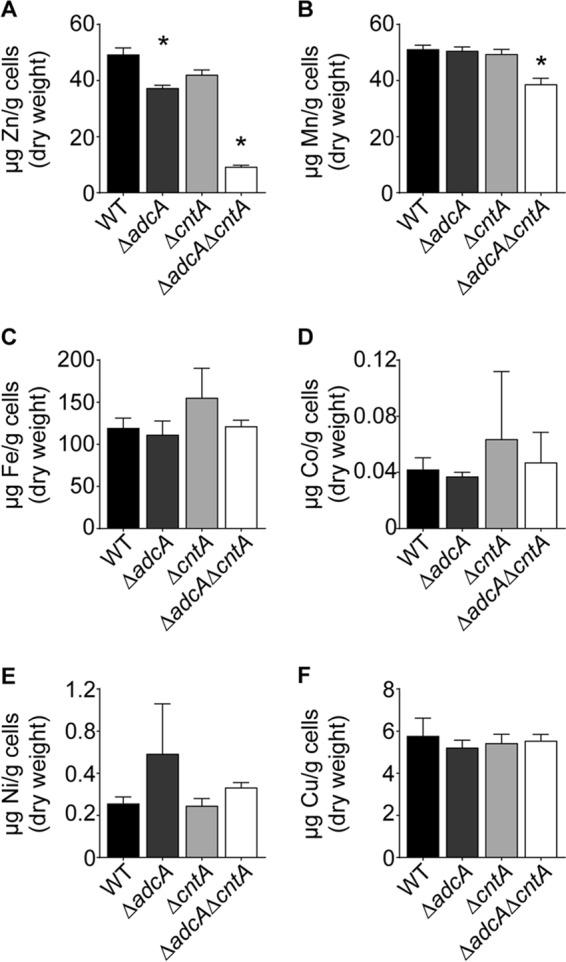
AdcABC and CntABCDF are Zn importers. Wild-type (WT) *S. aureus* and Δ*adcA*, Δ*cntA*, and Δ*adcA* Δ*cntA* mutants were grown in rich medium, and the intracellular metal content was analyzed utilizing ICP-MS. (A) Zn; (B) Mn; (C) Fe; (D) Co; (E) Ni; (F) Cu. *n* ≥ 2 or more. Error bars in all panels show standard errors of the means. *, *P* < 0.05 compared to wild type via one-way analysis of variance with Dunnett’s posttest.

Whole-cell metal accumulation was then assessed to directly determine the contribution of Adc and Cnt pathways to *S. aureus* metal uptake. Inductively coupled plasma mass spectrometry (ICP-MS) analysis of wild-type *S. aureus* and Δ*adcA*, Δ*cntA*, and Δ*adcA* Δ*cntA* mutants revealed a significant decrease in Zn accumulation by Δ*adcA* and Δ*adcA* Δ*cntA* strains (Fig. 2A). A modest reduction in Mn accumulation was also observed in the double mutant (Fig. 2B). However, this small reduction is unlikely to be contributing to the observed growth phenotype of the double mutant as the addition of Mn further exacerbated the growth defect of the Δ*adcA* Δ*cntA* strain (Fig. 1C). No change in the accumulation of Fe, Co, Ni, or Cu was observed with any of the mutant strains (Fig. 2C to F). Collectively, these results directly show that AdcABC and CntABCDF contribute to *S. aureus* Zn import. Further, CntABCDF, which has not been previously associated with Zn acquisition, does not functionally contribute to the uptake of metal ions other than Zn in *S. aureus*.

### Zinc acquisition by the Cnt transporter facilitates competition with calprotectin.

**FIG 3:**
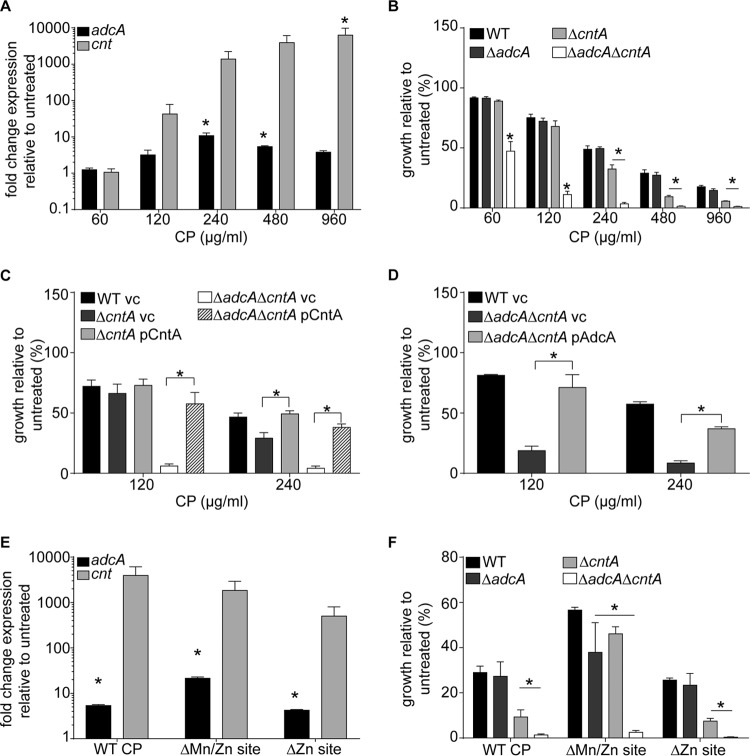
The CntABCDF importer enables *S. aureus* to resist CP-imposed starvation. (A) Wild-type *S. aureus* containing either the P_*adcA*_-YFP or the P_*cnt*_-YFP reporter plasmid was grown in the presence of various concentrations of CP, and fluorescence was assessed. *n* ≥ 3. *, *P* < 0.05 compared to untreated bacteria via one-way analysis of variance with Dunnett’s posttest. (B) Wild-type (WT) *S. aureus* and *ΔadcA*, *ΔcntA*, and *ΔadcA ΔcntA* mutants were incubated in the presence of increasing concentrations of CP, and growth was assessed by measuring optical density (OD_600_). *, *P* < 0.05 compared to wild type at the same CP concentration via two-way analysis of variance with Dunnett’s posttest. *n* ≥ 3. (C and D) Wild-type *S. aureus* and *ΔadcA ΔcntA* and *ΔcntA* mutants containing the indicated plasmids (vc, vector control) were incubated in the presence of CP, and growth was assessed by measuring optical density. *n* ≥ 3. *, *P* < 0.05 for the indicated comparison via two-way analysis of variance with Tukey’s posttest. (E) Wild-type *S. aureus* containing either the P_*adcA*_-YFP or the P_*cnt*_-YFP reporter plasmid was incubated in the presence or absence of 480 μg/ml of either wild-type CP, the ΔMn/Zn site mutant, or the ΔZn site mutant, and fluorescence was assessed. *n* ≥ 3. *, *P* < 0.05 compared to expression in the absence of CP via one-way analysis of variance with Dunnett’s posttest. (F) Wild-type *S. aureus* and *ΔadcA*, *ΔcntA*, and *ΔadcA ΔcntA* mutants were incubated in the presence or absence of 480 μg/ml of either WT CP, the ΔMn/Zn site mutant, or the ΔZn site mutant, and growth was assessed by measuring optical density (OD_600_). *n* ≥ 3. *, *P* < 0.05 compared to wild type via two-way analysis of variance with Dunnett’s posttest. Error bars in all panels show standard errors of the means.

We then sought to delineate the relative contribution of the two systems to resisting CP-imposed Zn starvation. Initially, we assessed how CP influenced transcription of the two Zn uptake systems. In response to CP, both systems were significantly upregulated (Fig. 3A). To assess the relative contributions of each system to Zn acquisition in the presence of CP, we then evaluated the growth of Δ*adcA*, Δ*cntA*, and Δ*adcA* Δ*cntA* mutants in the presence of CP. Consistent with our earlier observations, the Δ*adcA* Δ*cntA* mutant showed greater sensitivity to CP than did wild-type *S. aureus* at all concentrations tested (Fig. 3B). Loss of CntA diminished the ability of *S. aureus* to grow relative to the wild type when CP concentrations exceeded 120 µg/ml. In contrast, the Δ*adcA* mutant was no more sensitive to CP than the wild type (Fig. 3B). Ectopic expression of *cntABCDF* in Δ*cntA* and Δ*adcA* Δ*cntA* mutants restored wild-type growth in the presence of CP (Fig. 3C). Plasmid-mediated expression of *adcA* in the Δ*adcA* Δ*cntA* mutants permitted growth similar to that of the Δ*cntA* mutant (Fig. 3D). Additionally, loss of any of the genes associated with the Cnt importer in the methicillin-resistant strain USA300 (JE2) increased the sensitivity of *S. aureus* to CP (Fig. S2A). To determine if the Cnt system was important for resisting host-imposed Mn and/or Zn starvation, we used CP variants that lack either the Zn site (ΔZn, which binds both Mn and Zn) or the Mn/Zn site (ΔMn/Zn, which binds only Zn) (20). Expression of both the *adcA* and the *cnt* loci was induced in the presence of either CP site mutant (Fig. 3E). Both Δ*cntA* and Δ*adcA* Δ*cntA* mutants were sensitive to both the ΔZn and the ΔMn/Zn CP binding site mutants, as both can bind Zn (Fig. 3F), indicating that the increased sensitivity of these strains is due to a reduced ability to compete for Zn. Unexpectedly, we observed that the Δ*adcA* mutant is more sensitive than wild-type *S. aureus* to the ΔMn/Zn site mutant. As the Δ*adcA* mutant is not more sensitive to wild-type CP or ΔZn CP, this result suggests that the Cnt system is a less effective Zn importer when other metals, which could block Zn transport, are freely available, as would occur in the presence of the ΔMn/Zn site mutant, which lacks the ability to restrict Mn availability (Fig. 3F). In summary, these data demonstrate that the Cnt system contributes more to resisting CP-mediated Zn restriction than AdcABC.

10.1128/mBio.01281-17.2 FIG S2  Loss of the Cnt system renders the methicillin-resistant strain USA300 (JE2) more sensitive to CP-imposed metal starvation. (A and B) Wild-type *S. aureus* and *ΔcntA*, *ΔcntB*, *ΔcntC*, *ΔcntD*, *ΔcntF*, *ΔcntK*, *ΔcntL*, and *ΔcntM* USA300 (JE2) derivatives were incubated in the presence of increasing concentrations of CP, and growth was assessed by measuring optical density (OD_600_). *, *P* < 0.05 compared to wild type at the same CP concentration via two-way analysis of variance with Dunnett’s posttest. *n* ≥ 3. Error bars show standard errors of the means. Download FIG S2, TIF file, 0.3 MB. Copyright © 2017 Grim et al. 2017 Grim et al. This content is distributed under the terms of the Creative Commons Attribution 4.0 International license.

### Staphylopine functions as a zincophore enabling *S. aureus* to compete with CP for Zn.

**FIG 4:**
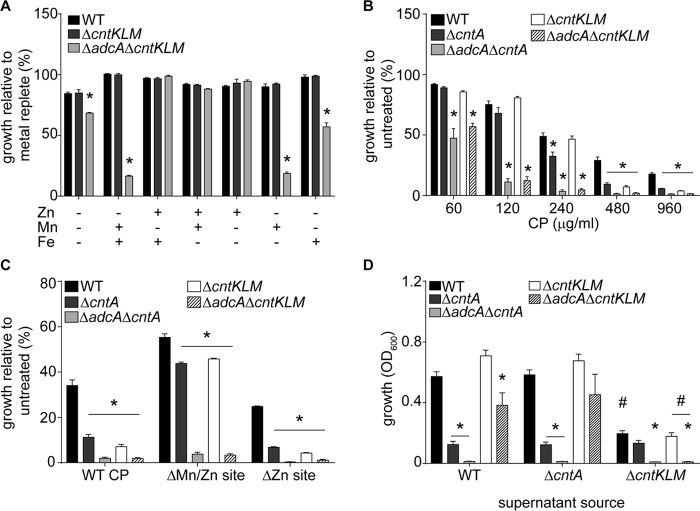
Staphylopine enables *S. aureus* to resist Zn starvation by functioning as a zincophore. (A) Wild-type (WT) *S. aureus* and *ΔcntKLM* and *ΔadcA ΔcntKLM* mutants were incubated in NRPMI supplemented with 25 μM ZnSO_4_, 25 μM MnCl_2_, and 25 μM FeSO_4_ as indicated, and growth was assessed by measuring optical density (OD_600_). (B) Wild-type *S. aureus* and *ΔcntA*, *ΔcntKLM*, *ΔadcA ΔcntA*, and *ΔadcA ΔcntKLM* mutants were incubated in various concentrations of CP, and growth was assessed by measuring optical density (OD_600_). (C) Wild-type *S. aureus* and the *ΔadcA ΔcntA* mutant were incubated in the presence or absence of 480 μg/ml of either wild-type CP, the ΔMn/Zn site mutant, or the ΔZn site mutant, and growth was assessed by measuring optical density (OD_600_). (D) Supernatant was harvested from wild-type *S. aureus* or the Δ*cntA* or Δ*cntKLM* mutant following growth in defined medium lacking Zn. The supernatant was then assessed for the ability to rescue the growth of wild-type *S. aureus* or Δ*cntA*, Δ*cntKLM*, *ΔadcA ΔcntA*, and *ΔadcA ΔcntKLM* mutants when incubated in the presence of CP. Growth in the presence of the various supernatants and CP was assessed by measuring the OD_600_. (A to D) *n* ≥ 3. *, *P* < 0.05 compared to wild type in the same supernatant treatment via two-way analysis of variance using Dunnett’s posttest. Error bars show standard errors of the means. (D) #, *P* < 0.05 compared to wild-type supernatant via two-way analysis of variance using Dunnett’s posttest.

Distinct from the direct metal ion recruitment mechanisms of the cluster A-I SBPs, the cluster C SBPs, employed by Opp/NikA ABC permeases, bind metal chelates in a process analogous to siderophore-iron acquisition systems. The *cnt* locus also encodes CntKLM and CntE, which produce and secrete, respectively, the broad-spectrum metallophore staphylopine (StP). Extracellular StP can then be imported by the CntABCDF transporter (46). Loss of StP, similar to that of CntA (Fig. 1C) (46), does not inhibit the growth of *S. aureus* in medium that has had the metal content reduced using conventional approaches (Fig. 4A) (46). Our results suggest that the presence of the Adc permease may have obscured a role for StP in Zn uptake. However, the promiscuity of Opp/NikA-type transporters (47–49) raises the possibility that StP may not be the metallophore involved in Zn acquisition. We sought to address this by evaluating the ability of an Δ*adcA* Δ*cntKLM* mutant to grow in Zn-depleted medium. Similarly to the Δ*adcA* Δ*cntA* strain, this mutant was severely attenuated for growth in Zn-restricted medium and profoundly more sensitive to CP (Fig. 4A and B). The addition of Zn, but not Fe or Mn, reversed the growth defect of the Δ*adcA* Δ*cntKLM* mutant (Fig. 4A). The growth defect of the Δ*adcA* Δ*cntKLM* mutant was also reversed by ectopic expression of *adcA* or *cntKLM* (Fig. S3). Similarly to the Δ*cntA* mutant, the Δc*ntKLM* mutant was also more sensitive to CP (Fig. 4B). This sensitivity was abolished by expression of *cntKLM* from a plasmid in the Δ*cntKLM* mutant (Fig. S3D). Similar results were also observed with USA300 (JE2) (Fig. S2B). The Δ*cntKLM* and Δ*adcA* Δ*cntKLM* mutants were also more sensitive than wild-type *S. aureus* to both CP variants (ΔZn CP and ΔMn/Zn CP), indicating that the observed growth defect is attributable to impaired Zn acquisition (Fig. 4C). Collectively, these results show that StP contributes to the ability of *S. aureus* to overcome host-imposed Zn starvation.

10.1128/mBio.01281-17.3 FIG S3  Ectopic expression of *adcA* and *cntKLM* reverses the growth defect of Δ*adcA* Δ*cntKLM* mutant. (A and B) Wild-type *S. aureus* and the Δ*adcA* Δ*cntKLM* mutant with the indicated plasmids (vc, vector control) were incubated in NRPMI supplemented with 25 μM MnCl_2_ and 25 μM FeSO_4_ in the presence and absence of 25 μM ZnSO_4_, and growth was assessed by measuring optical density (OD_600_). *n* = 3. *, *P* < 0.05 for the indicated comparison via two-way analysis of variance using Tukey’s posttest. (C and D) Wild-type *S. aureus* and *ΔadcA ΔcntKLM* and *ΔcntKLM* mutants containing the indicated plasmids were incubated in the presence of CP, and growth was assessed by measuring optical density (OD_600_). *n* = 3. *, *P* < 0.05 for the indicated comparison via two-way analysis of variance with Tukey’s posttest. (A to D) Error bars show standard errors of the means. Download FIG S3, TIF file, 12 MB. Copyright © 2017 Grim et al. 2017 Grim et al. This content is distributed under the terms of the Creative Commons Attribution 4.0 International license.

Next, we evaluated if StP is promoting growth in Zn-restricted environments by functioning as a zincophore. If StP is a zincophore, it should function in *trans* and be dependent on the ABC permease CntABCDF. To evaluate this possibility, supernatants harvested from Zn-starved wild-type *S. aureus* and Δ*cntA* and Δ*cntKLM* mutants were tested for their ability to rescue the growth of various staphylococcal mutants. The supernatants harvested from wild-type *S. aureus* and the Δ*cntA* mutant rescued the growth of both Δ*cntKLM* and *ΔadcA* Δ*cntKLM* strains but not strains lacking the SBP CntA (Fig. 4D and S4). These data indicate that CntABCDF and StP function together to promote resistance to CP-mediated Zn starvation. Supernatant harvested from a strain deficient in StP synthesis, the Δ*cntKLM* mutant, was unable to rescue the growth of any strain tested (Fig. 4D and S4). Collectively, these results demonstrate that StP functions as a zincophore, enhancing the ability of *S. aureus* to compete with CP for Zn.

10.1128/mBio.01281-17.4 FIG S4  Staphylopine enables *S. aureus* to resist Zn starvation by functioning as a zincophore. (A to E) Supernatant was harvested from wild-type *S. aureus* or the Δ*cntA* or Δ*cntKLM* mutant following growth in defined medium lacking Zn. The supernatant was then assessed for the ability to rescue the growth of wild-type *S. aureus* or Δ*cntA*, Δ*cntKLM*, *ΔadcA ΔcntA*, and *ΔadcA ΔcntKLM* mutants when incubated in the presence of CP. Growth in the presence of the various supernatants and CP was assessed by measuring the OD_600_. *, *P* < 0.05 compared to wild type via two-way analysis of variance using Dunnett’s posttest. Error bars show standard errors of the means. *n* = 3. Download FIG S4, TIF file, 0.5 MB. Copyright © 2017 Grim et al. 2017 Grim et al. This content is distributed under the terms of the Creative Commons Attribution 4.0 International license.

### The Cnt-StP system is the dominant Zn importer utilized during systemic infection.

**FIG 5:**
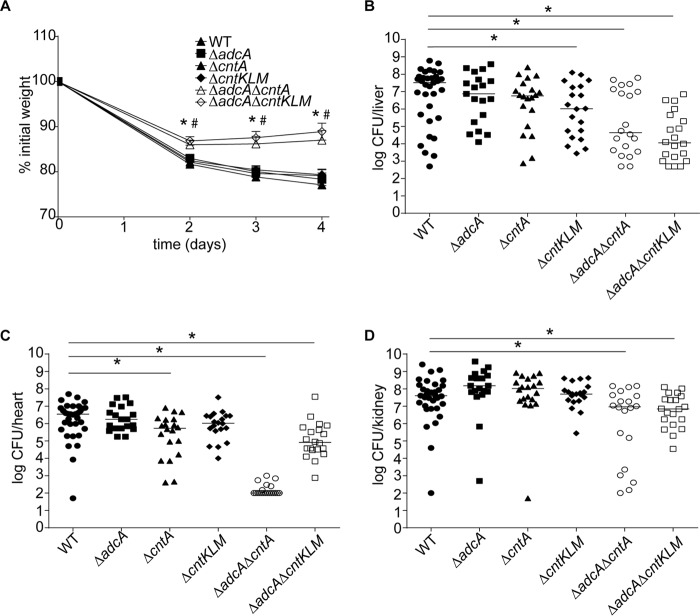
The Cnt-StP system is used to resist host-imposed metal starvation during infection. C57BL/6 mice were retroorbitally infected with ~1 × 10^7^ CFU of wild-type (WT) *S. aureus* or *ΔadcA*, Δ*cntA*, Δ*cntKLM*, Δ*adcA* Δ*cntA*, or Δ*adcA* Δ*cntKLM* mutant, and weight (A) and bacterial burdens in the liver (B), heart (C), and kidneys (D) were assessed. (A) * and #, *P* < 0.05 relative to mice infected with wild-type *S. aureus* for Δ*adcA* Δ*cntA* and Δ*adcA* Δ*cntKLM* mutants, respectively, via two-way analysis of variance with Dunnett’s posttest. Error bars show standard errors of the means. (B to D) *, *P* < 0.05 compared to mice infected with wild-type *S. aureus* via Mann-Whitney U test.

To evaluate the contributions of AdcABC, CntABCDF, and StP to staphylococcal infection, a systemic retroorbital infection model was used. For these studies, C57BL/6 mice were infected with either wild-type *S. aureus* or the Δ*adcA*, Δ*cntA*, Δ*cntKLM*, Δ*adcA* Δ*cntA*, or Δ*adcA* Δ*cntKLM* mutant (Fig. 5A to D). Mice infected with the double mutants lost significantly less weight than those infected with wild-type *S. aureus* or the single mutants (Fig. 5A). Consistent with the CP growth assays, mice infected with the Δ*adcA* strain had bacterial burdens comparable to those infected with the wild type. Compared to the wild type, the Δ*cntA* mutant showed a significant reduction in bacterial burden in the heart, while the Δ*cntKLM* mutant had a reduced burden in the liver. Both Δ*adcA* Δ*cntA* and Δ*adcA* Δ*cntKLM* mutants had reduced bacterial burdens in the liver, heart, and kidneys (Fig. 5B to D). Together, these results indicate that while both Zn import pathways can contribute to the ability of *S. aureus* to cause disease, the Cnt-StP system is sufficient to facilitate Zn acquisition during infection.

### Staphylopine analogs are widely distributed in bacteria.

**FIG 6:**
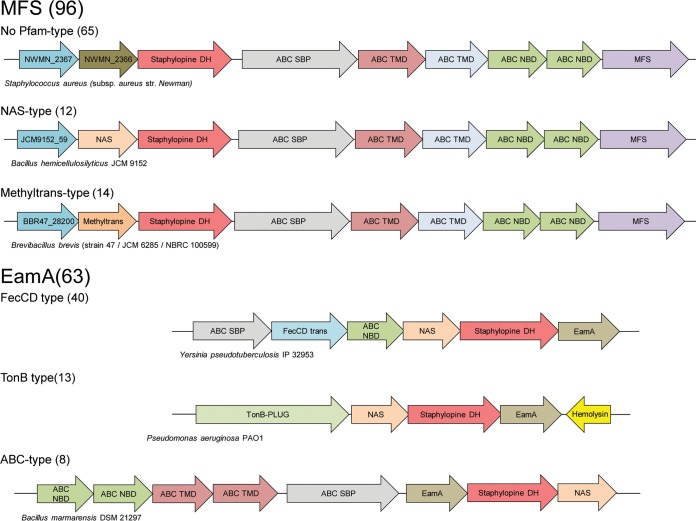
Bacteria possess a variety of StP-like synthesis loci. Diagrams of the 6 families of StP synthesis loci identified by neighborhood network analysis. See Table S1 in the supplemental material for a complete list of the loci identified. Abbreviations: NBD, nucleotide-binding domain; TMD, transmembrane domain; DH, dehydrogenase; NAS, nicotianamine synthase.

Having established a role for StP in the pathogenesis of *S. aureus*, we then investigated the distribution of the StP synthesis locus using genome neighborhood network (GNN) analysis. StP is produced by CntK, a histidine racemase; CntL, an enzyme with low similarity to nicotianamine synthase; and CntM, which attaches a pyruvate moiety to the StP precursor. CntM, the only member of a Pfam/InterPro protein family, was used to anchor the analysis (Fig. 6 and Table S1). The CntM InterPro family (IPR016935, 184 nonredundant, nonobsolete, queriable members) contains ~90 unique species. Using this data set, 92% (170/184) of the time immediately adjacent (median gene distance of ±1 open reading frame [ORF]) to CntM was a gene belonging to one of three categories. Fifty percent of the time (85/170), a member of the nicotianamine synthase Pfam (PF03059) was immediately adjacent to the CntM homolog. Nine percent (15/170) of the time, a member of the methyltranferase_31 Pfam (PF13847) was next to the CntM homolog. The remaining 41% (70/170) of the encoded proteins belonged to no Pfam. BLAST analysis of the latter group revealed that 70% of the sequences shared a high degree of similarity (50% sequence identity or greater) with CntL from *S. aureus*. Surprisingly, CntK, CntL homologs, and the methyltransferase_31 Pfam family are largely restricted to the *Firmicutes*. Predicted importer and efflux system were associated with all of the synthesis clusters. While there was variability in the importer associated with the synthesis loci, the putative efflux pump belonged to either the major facilitator superfamily (MFS) or the EamA family of transporters. Notably, the MFS-containing loci were primarily associated with CntABCDF importer homologs and divergent staphylopine synthesis machinery, while the EamA loci were associated with a variety of potential importers but contained conserved core synthesis machinery. Additionally, for at least 20 of the genomes analyzed, additional enzymes were encoded proximal to *cntLM* that may result in the production of modified StP-like molecules. Collectively, these observations suggest that a diverse collection of StP analogs is present in a variety of Gram-positive and Gram-negative organisms.

10.1128/mBio.01281-17.5 TABLE S1  Nonredundant list of species that possess staphylopine-like synthesis loci. Download TABLE S1, PDF file, 0.03 MB. Copyright © 2017 Grim et al. 2017 Grim et al. This content is distributed under the terms of the Creative Commons Attribution 4.0 International license.

## DISCUSSION

During infection, nutritional immunity severely restricts the bioavailability of the essential nutrient Zn (8, 12). Despite this challenge, successful pathogens, such as *S. aureus*, remain capable of causing devastating disease. The success of *S. aureus* and other invaders is mediated by an ability to compete with the host for Zn (13, 20). Our work shows that *S. aureus* possesses two distinct types of ABC permeases, AdcABC and CntABCDF, involved in Zn acquisition. AdcABC is homologous to ABC permeases associated with direct recruitment of Zn. CntABCDF belongs to the NikA/Opp family of ABC permeases, which have not previously been associated with Zn acquisition. Our work revealed that CntABCDF functions in conjunction with the recently identified broad-spectrum metallophore StP to specifically promote Zn acquisition. These results indicate that, although StP can bind a variety of metals *in vitro*, it functions as a staphylococcal zincophore. Collectively, our findings conclusively establish the existence of a new class of bacterial Zn ABC importers.

Many bacteria, including *S. aureus*, contain an array of distinct Fe and Mn acquisition systems. Due to their overlapping functions, the disruption of multiple metal transporters is frequently required to observe a phenotype (12, 50–53). The presence of multiple Zn uptake pathways in bacteria is also well established, but typically these transporters all belong to the Znu/Adc ABC permease family (54, 55). Loss of CntA or StP does not impair the ability of *S. aureus* to grow or to obtain Zn in medium rendered Zn depleted using conventional approaches. Despite the transcriptional responsiveness of the *cnt* locus to Zn abundance, this led to the conclusion that the Cnt-StP system was not a Zn importer (45, 46). At the time that the studies were conducted, there was a paucity of data on the Zn acquisition systems possessed by *S. aureus*. Identification of the AdcABC Zn importer in *S. aureus* suggests that the prior lack of a Zn-associated phenotype in CntA and StP single mutants is due to overlapping functions. The observations that strains lacking both the Cnt-StP system and Adc permease have major growth defects in Zn-limited medium and selectively fail to accumulate Zn demonstrate that these systems serve as the major Zn importers of the pathogen. Consequently, this work defines the Cnt-StP system as the founding member of a new class of Zn importers and expands the use of bacterially produced metallophores beyond Fe.

StP is a broad-spectrum metallophore, and transport assays following growth in Zn-depleted medium have demonstrated that the Cnt-StP system can import Cu, Co, and Ni (45, 46). This raises the possibility that the system could contribute to the ability of *S. aureus* to obtain these metals. However, metal content analyses revealed only defects in Zn accumulation. Additionally, high-affinity metal importers are typically regulated by the cellular abundance of their cognate metal (11, 43, 56). Our work and that of others (45) have shown that Co and Ni abundance does not influence the expression of the *cnt* locus. Furthermore, *S. aureus* possess two bona fide Ni transporters, the loss of which does reduce accumulation of Ni (57). With respect to Cu accumulation, due to its potent toxicity, even low levels of this metal are sufficient to induce the expression of dedicated efflux pumps. As such, it is unlikely that *S. aureus* actively accumulates this metal (58). Taken together, the balance of evidence indicates that Co, Ni, and Cu are not physiological substrates of the Cnt-StP system. Distinctly from Co and Ni, our work and that of others (59) suggest that the Cnt-StP system is modestly responsive to Mn and Fe. However, these metals exert an influence on transcription only in the absence of Zn. This suggests that Zn abundance is the principal regulatory factor controlling expression of the system. Further supporting a role in Zn transport is the observation that Mn and Fe supplementation suppresses growth of the Δ*adcA* Δ*cntA* mutant. Collectively, these data indicate that the physiological role of the Cnt-StP system is as a Zn acquisition pathway.

StP synthesis loci are present in numerous pathogens, including multiple staphylococcal, *Yersinia*, and *Pseudomonas* species, suggesting that StP analogs may play an important role in the pathogenesis of several microbes. Intriguingly, while all of the putative synthesis loci contain genes encoding CntM and CntL, 45% lacked a gene encoding a CntK homolog, suggesting that both d- and l-isomers of StP are produced, depending on the species. Additional genes that appear to encode small-molecule-modifying enzymes were associated with some of the StP synthesis loci. These observations suggest that bacteria produce an array of diverse metal chelators that are related to but are distinct from StP. This inference is supported by the observation that importers that are not homologous to the CntABCDF permease are associated with StP loci in other bacteria. Microbes are known to steal siderophores produced by other organisms; thus, the production of StP variants may serve as a mechanism to prevent their use by other microorganisms (32, 34, 60–62). It is also tempting to speculate that this diversity may serve as a mechanism to prevent the host from binding the zincophore produced by a pathogen, akin to the production of modified siderophores that evade binding by the host immune effector lipocalin (63–65).

In *S*. Typhimurium and *A. baumannii*, loss of the AdcABC family importer severely impairs their ability to compete with CP for Zn and cause infection (17, 23). Loss of AdcABC permeases also impairs the ability of *Vibrio cholerae*, *Streptococcus pneumoniae*, *Listeria monocytogenes*, and other pathogens to cause infection (38, 55, 66, 67). In *P. aeruginosa*, loss of the AdcA homolog ZnuA modestly diminishes the ability of the bacterium to grow in the presence of CP and in Zn-limited medium (24, 68). Differing from these pathogens, loss of AdcABC alone does not diminish the ability of *S. aureus* to grow in the presence of CP or cause disease. However, the Cnt-StP system is critical to the ability of *S. aureus* to resist CP-imposed Zn starvation. In combination with the virulence defects associated with Δ*cntKLM*, Δ*cntA*, and Δ*cntE* (Fig. 5A to D) (59), these results indicate that this zincophore-based importer is the main system used by *S. aureus* to compete with the host for Zn during infection. Unfortunately, mice lacking CP, in the context of a *S. aureus* infection, do not have defects in Zn sequestration, preventing this idea from being directly tested as has been done for the staphylococcal Mn transporters (8, 12). Further supporting this supposition is the observation that the virulence defects of strains lacking CntKLM or CntA are exacerbated by concurrent loss of AdcA. Similarly to *S. aureus*, *Yersinia pestis* lacking the Adc permease does not have a virulence defect (43). This observation is potentially explained by the presence of an StP synthesis locus in *Y. pestis*, which is Zur regulated (69). The presence of an StP analog could also explain the modest phenotypes of *P. aeruginosa* strains lacking the Znu system. While the *P. aeruginosa* StP analog is reported to be a siderophore, it is regulated by Zur, which strongly suggests a role in Zn acquisition (68, 70). These observations suggest that StP analogs and their cognate transporters likely contribute to the ability of multiple pathogens to compete with the host for Zn.

The identification of the Zn acquisition systems employed by *S. aureus* offers new opportunities to disrupt the ability of pathogens to compete with the host for Zn. The widespread prevalence of the StP synthesis machinery in both Gram-positive and Gram-negative pathogens suggests that information gained by studying these systems will provide critical insight into how numerous pathogens circumvent nutritional immunity.

## MATERIALS AND METHODS

### Ethics statement.

All animal experiments were approved by the University of Illinois at Urbana-Champaign Institutional Animal Care and Use Committee (IACUC license number 15059) and performed according to NIH guidelines, the Animal Welfare Act, and U.S. federal law.

### Bacterial strains.

For routine overnight cultures, *S. aureus* strains were inoculated into 5 ml of tryptic soy broth (TSB) in 15-ml conical tubes and grown at 37°C on a roller drum. To preculture bacteria in limited-metal environments, overnight growth was performed in 5 ml of Chelex-treated RPMI medium (NRPMI) supplemented with 1% Casamino Acids, 1 mM MgCl_2_, 100 μM CaCl_2_, and 1 μM FeCl_2_ in 15-ml conical tubes and bacteria were grown at 37°C on a roller drum. *S. aureus* Newman and derivatives were used for all experiments, unless otherwise noted. The Δ*cntA* and Δ*cntKLM* mutants were generated by amplifying the 5′ and 3′ flanking regions of the genes using the indicated primers (see Table S2 in the supplemental material). These fragments were then cloned into pKOR1, and the deletions were generated using allelic replacement, as previously described (71). The *adcA*::*erm* mutant was generated by phage transducing the allele from USA300 (JE2) *adcA*::*erm* into Newman via Φ85 phage. For complementation constructs, the *cntA*, *adcA*, and *cntKLM* coding sequences were amplified using the indicated primers (Table S2). The *cntA* and *cntKLM* coding sequences were cloned into pRMC2, which contains an anhydrotetracycline-inducible promoter (72). The *adcA* coding sequence was cloned into pOS1 under the control of the *lgt* promoter. For the fluorescent reporters, the promoters of the *cnt* operon and *adcA* were cloned into the yellow fluorescent protein (YFP)-containing vector pAH5 (73). All constructs were verified by sequencing, and all mutants were confirmed to be hemolytic. See Tables S3 and S4 for a full list of the strains and plasmids used in this study, respectively.

10.1128/mBio.01281-17.6 TABLE S2  Primers used in this study. Download TABLE S2, DOCX file, 0.01 MB. Copyright © 2017 Grim et al. 2017 Grim et al. This content is distributed under the terms of the Creative Commons Attribution 4.0 International license.

10.1128/mBio.01281-17.7 TABLE S3  Strains used in this study. Download TABLE S3, DOCX file, 0.01 MB. Copyright © 2017 Grim et al. 2017 Grim et al. This content is distributed under the terms of the Creative Commons Attribution 4.0 International license.

10.1128/mBio.01281-17.8 TABLE S4  Plasmids used in this study. Download TABLE S4, DOCX file, 0.02 MB. Copyright © 2017 Grim et al. 2017 Grim et al. This content is distributed under the terms of the Creative Commons Attribution 4.0 International license.

### NRPMI growth assays.

For growth assays using NRPMI, following overnight growth in TSB, the cultures were back-diluted 1:50 in 5 ml of TSB for 1 h at 37°C. The cultures were then diluted 1:100 in 96-well round-bottom plates containing 100 μl of NRPMI (12) containing 100 μM CaCl_2_ and 1 mM MgCl_2_ and combinations of 25 μM ZnSO_4_, MnCl_2_, FeSO_4_, CoCl_2_, or NiSO_4_. Cultures were incubated at 37°C with shaking at 180 rpm, and growth was assessed by measuring the optical density at 600 nm (OD_600_). For complementation experiments, the cultures were also supplemented with 10 μg/ml of chloramphenicol. Cultures in experiments using pRMC2 were also supplemented with 10 ng/ml of anhydrotetracycline.

### Calprotectin growth assays.

CP growth assays were performed as previously described with minor modifications (10, 13, 20). Briefly, overnight cultures were back-diluted 1:50 in 5 ml of TSB for 1 h at 37°C. The cultures were then back-diluted 1:100 in 96-well round-bottom plates containing 100 μl of growth medium, which consisted of 38% TSB and 62% calprotectin buffer (20 mM Tris, pH 7.5, 100 mM NaCl, 3 mM CaCl_2_, 10 mM β-mercaptoethanol) and was supplemented with 1 μM MnCl_2_ and 1 μM ZnSO_4_. Cultures were incubated at 37°C with shaking at 180 rpm, and growth was assessed by measuring the optical density at 600 nm (OD_600_). For complementation experiments, bacteria were back-diluted 1:50 in 5 ml of TSB at 37°C for 2 h and supplemented with 10 μg/ml of chloramphenicol. For the experiments using the USA300 (JE2) Δ*cntK*, Δ*cntL*, and Δ*cntM* mutants, the bacteria were grown overnight in TSB and then washed with phosphate-buffered saline (PBS). For these mutants, the growth medium consisted of 38% defined medium (2.6×) and 62% CP buffer (20 mM Tris, pH 7.5, 100 mM NaCl, 3 mM CaCl_2_, 10 mM β-mercaptoethanol) (13). The defined medium (2.6×) consisted of 0.5 g/liter NaCl, 1.0 g/liter NH_4_Cl, 2 g/liter KH_2_PO_4_, 7 g/liter Na_2_HPO_4_, 0.228 µg/liter biotin, 0.228 mg/liter nicotinic acid, 0.228 mg/liter pyridoxine-HCl, 0.228 mg/liter thiamine-HCl, 0.114 mg/liter riboflavin, 0.684 mg/liter calcium pantothenate, 0.104 g/liter phenylalanine, 0.078 g/liter isoleucine, 0.130 g/liter tyrosine, 0.053 g/liter cysteine, 0.260 g/liter glutamic acid, 0.026 g/liter lysine, 0.182 g/liter methionine, 0.078 g/liter histidine, 0.026 g/liter tryptophan, 0.234 g/liter leucine, 0.234 g/liter aspartic acid, 0.182 g/liter arginine, 0.078 g/liter serine, 0.156 g/liter alanine, 0.078 g/liter threonine, 0.130 g/liter glycine, 0.208 g/liter valine, and 0.026 g/liter proline. This was then supplemented with 1.3% glucose. Prior to bacterial growth, the medium was supplemented with 1 μM MnCl_2_, 1 μM ZnSO_4_, 1 μM FeSO_4_, and 2.3 mM MgSO_4_. For supernatant rescue assays, the bacteria were grown in Chelex-treated defined medium. Prior to bacterial growth, the medium was supplemented with 1 μM MnCl_2_, 1 μM FeSO_4_, and 2.3 mM MgSO_4_. Bacterial cultures were grown to late exponential phase, and the supernatants were harvested following centrifugation of the cultures. The supernatant was collected, concentrated approximately 15-fold under reduced pressure using a rotary evaporator, and sterile filtered using a 0.22-μm polyether sulfone (PES) membrane filter. For CP assays using concentrated supernatant, the medium consisted of 19% 2× TSB, 62% CP buffer (20 mM Tris, pH 7.5, 100 mM NaCl, 1 mM CaCl_2_, 10 mM β-mercaptoethanol), and 19% supernatant. CP was purified as previously described (10, 12).

### Expression analysis.

The expression of *adcA* and the *cnt* locus was assessed using a YFP-promoter fusion (excitation and emission wavelengths of 505 nm and 535 nm, respectively) as previously described (30). For assays using NRPMI, the bacteria were precultured overnight in NRPMI as described above. They were then back-diluted 1:100 in a 96-well plate containing 100 μl of NRPMI supplemented with 100 μM CaCl_2_ and 1 mM MgCl_2_ and various concentrations of ZnSO_4_, MnCl_2_, FeSO_4_, CoCl_2_, and NiSO_4_. For assays assessing expression in the presence of CP, bacteria were grown as described above for CP growth assays.

### Animal infections.

All animal experiments were performed as previously described (8, 10, 12, 13, 30). Nine-week-old female C57BL/6 mice were injected retroorbitally with 100 μl of 1 × 10^8^ CFU/ml of bacteria suspended in sterile PBS. Infection was allowed to proceed for 4 days. On day 4 postinfection, mice were sacrificed and their livers, kidneys, and hearts were harvested. Bacterial burdens were assessed by dilution plating.

### Elemental analysis.

Whole-cell metal accumulation of *S. aureus* strains was performed using the growth parameters described for the CP growth inhibition assay. Bacteria were harvested during log-phase growth (OD_600_ of ~0.1) and then washed by resuspension and centrifugation at 3,700 × *g* for 10 min, twice with 100 mM EDTA and then twice with sterile double-distilled water (ddH_2_O). They were then suspended in 1 ml of sterile water, and a small aliquot was taken to determine the CFU. The bacteria were then centrifuged, and the supernatant was removed. Bacterial pellets were desiccated at 96°C overnight. The dry weight of cells was measured, and the pellets were resuspended in 35% HNO_3_ and boiled at 95°C for 1 h prior to removal of debris by centrifugation. Samples were diluted to a final concentration of 3.5% HNO_3_ and analyzed by inductively coupled plasma mass spectrometry (ICP-MS) on an Agilent 7500cx ICP-MS (Adelaide Microscopy, University of Adelaide) as described previously (74, 75).

### Bioinformatics.

Sequence similarity networks (SSNs) were generated according to the procedure described elsewhere (76, 77). Briefly, the InterPro family IPR016935, of which CntM is a member, was used as input for the EFI-EST web tool (http://efi.igb.illinois.edu/efi-est/). The initial SSN was generated with an alignment score threshold corresponding to ~50% sequence identity. To identify the genomic cooccurrence between members of InterPro family IPR016935 and other putative staphylopine biosynthetic genes (*cntL* and *cntK*) and transporters, the single SSN cluster was uploaded to the EFI-GNT web tool (http://efi.igb.illinois.edu/efi-gnt/) using the default “neighborhood size” (±10 genes) and an “input % co-occurrence lower limit” of 5%. The resulting genome neighborhood network (GNN) quantifies the frequency with which gene products (functionally identified by the Pfam families of the encoded proteins) are encoded proximal to the genes for members of the SSN query cluster. Given the tendency for genes of functionally linked proteins to be proximal to one another in bacterial genomes, the GNN can be used to infer functional relationships between the query and neighboring proteins. Neither CntL nor CntK is a member of Pfam families; therefore, to identify homologs for these sequences, a protein BLAST search was performed separately for CntL and CntK (http://www.uniprot.org/blast/; generated using default parameters and BLOSUM62 matrix), and the resulting list was searched against the complete list of genome proximal sequences returned in the IPR016935 GNN. Cytoscape v3.2.0 (78) was used for visualization and analysis of the SSN and GNN.

### Quantification and statistical analysis.

All statistical analyses were performed using GraphPad Prism version 6. The specific statistical test used is indicated in each figure legend.
