# Supplementary figures and images for: The Metallophore Staphylopine Enables Staphylococcus aureus To Compete with the Host for Zinc and Overcome Nutritional Immunity
Source: mBio. 2017 Oct 31;8(5):e01281-17. doi: 10.1128/mBio.01281-17 (PMC5666155; doi:10.1128/mBio.01281-17)

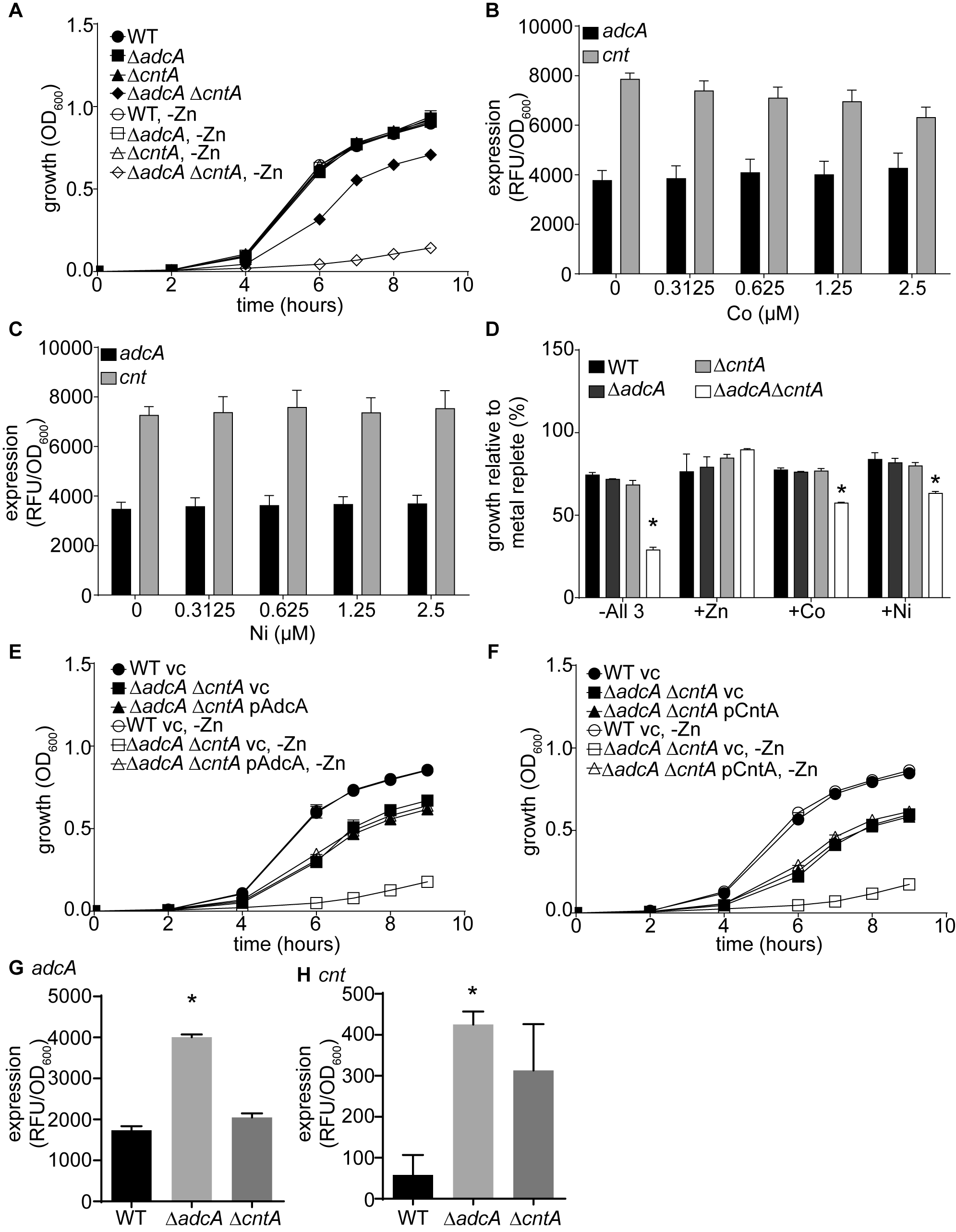

Supplement: FIG S1 [file mbo005173560sf1.tif]

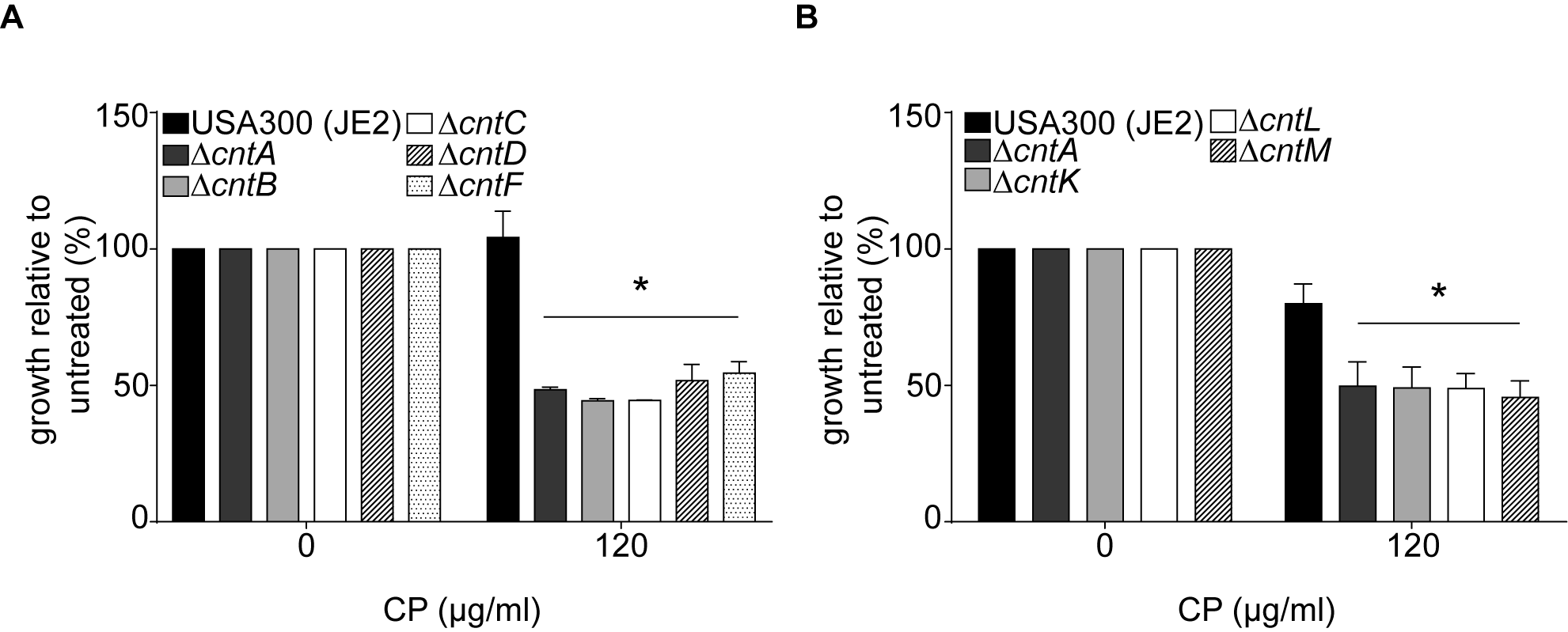

Supplement: FIG S2 [file mbo005173560sf2.tif]

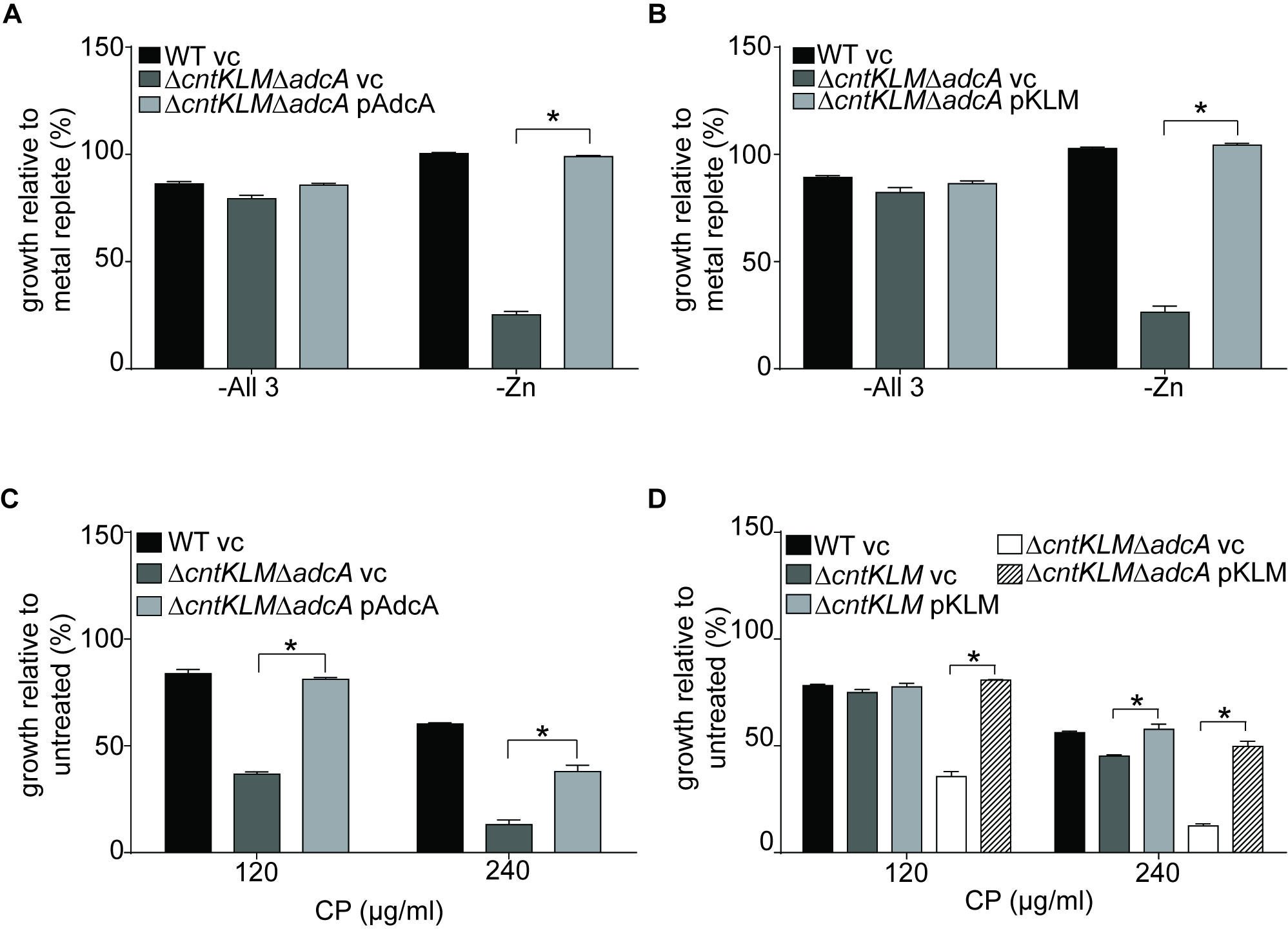

Supplement: FIG S3 [file mbo005173560sf3.tif]

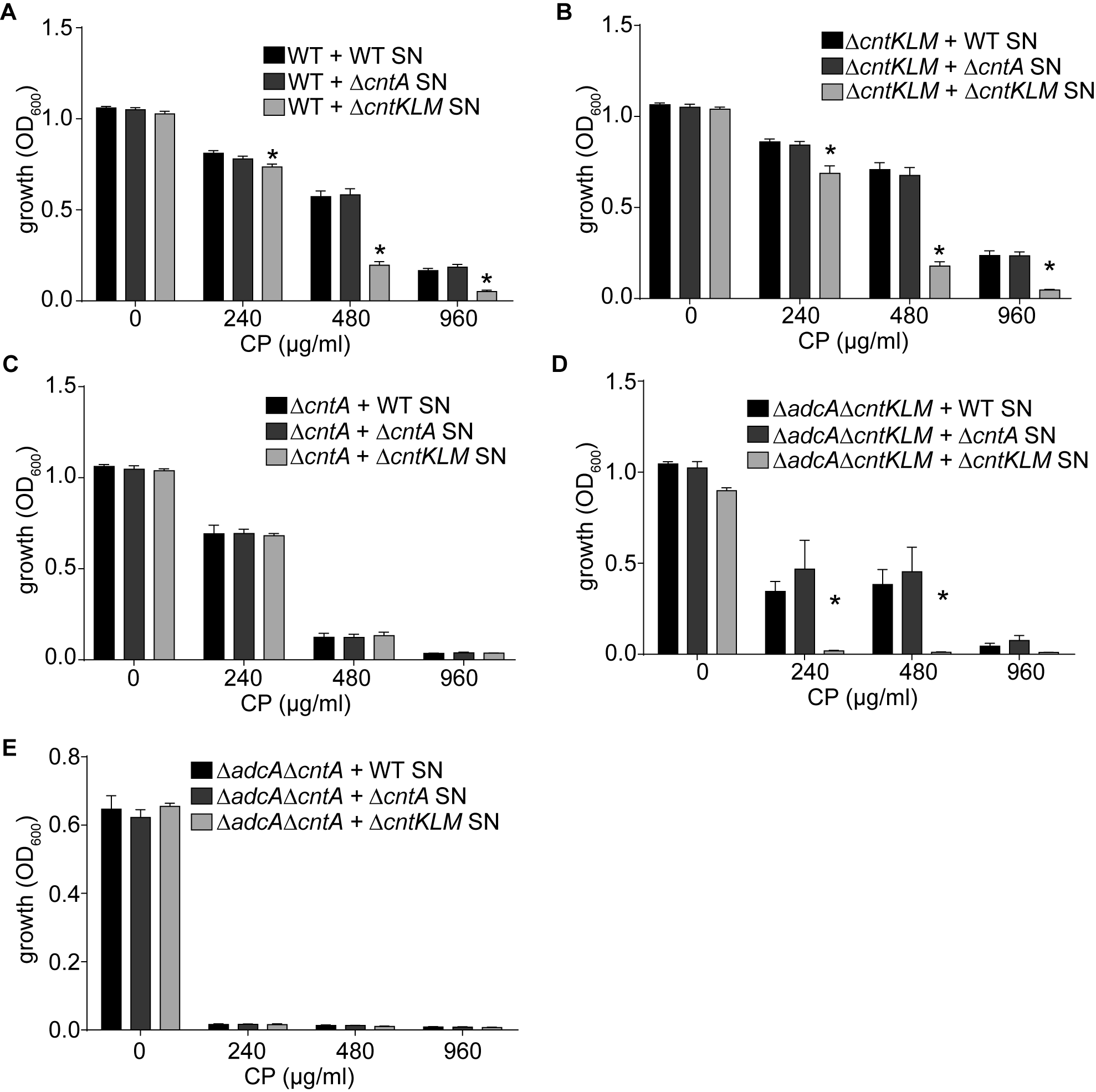

Supplement: FIG S4 [file mbo005173560sf4.tif]
