# Supplementary material for: The Metallophore Staphylopine Enables Staphylococcus aureus To Compete with the Host for Zinc and Overcome Nutritional Immunity
Source: mBio. 2017 Oct 31;8(5):e01281-17. doi: 10.1128/mBio.01281-17 (PMC5666155; doi:10.1128/mBio.01281-17)
Supplement: TABLE S1 [file mbo005173560st1.pdf]

Organism (UniProt ID, to distinguish between species)	Pathway type	Pathway subtype
#unknown (A0A0G9N3B2)	EamA	FecCD
#unknown (A0A0M2W2B8)	EamA	FecCD
Arsenophonus nasoniae (son-killer infecting Nasonia vitripennis)	EamA	FecCD
Clostridium argentinense CDC 2741	EamA	FecCD
Fusobacterium nucleatum CTI-1	EamA	FecCD
Fusobacterium nucleatum subsp. animalis 7_1	EamA	FecCD
Fusobacterium ulcerans 12-1B	EamA	FecCD
Fusobacterium ulcerans ATCC 49185	EamA	FecCD
Fusobacterium varium ATCC 27725	EamA	FecCD
Klebsiella oxytoca (A0A168L0N9)	EamA	FecCD
Serratia liquefaciens FK01	EamA	FecCD
Serratia marcescens (A0A0G8B6E7)	EamA	FecCD
Serratia marcescens (A0A0P0QDX9)	EamA	FecCD
Serratia marcescens (A0A0U6G5J4)	EamA	FecCD
Serratia marcescens (A0A1C3HCW1)	EamA	FecCD
Serratia marcescens subsp. marcescens (A0A1C0CMK1)	EamA	FecCD
Serratia nematodiphila DZ0503SBS1	EamA	FecCD
Serratia sp. YD25	EamA	FecCD
Vibrio caribbeanicus ATCC BAA-2122	EamA	FecCD
Vibrio crassostreae (A0A0T7DF42)	EamA	FecCD
Vibrio crassostreae 9CS106	EamA	FecCD
Vibrio neptunius (A0A0F4PDU9)	EamA	FecCD
Vibrio nigripulchritudo (U4KI97)	EamA	FecCD
Vibrio nigripulchritudo FTn2	EamA	FecCD
Vibrio sp. J2-29	EamA	FecCD
Vibrio sp. J2-4	EamA	FecCD
Yersinia intermedia (A0A0U1ENU9)	EamA	FecCD
Yersinia pestis (Q0WH68)	EamA	FecCD
Yersinia pestis (Q8CKU7)	EamA	FecCD
Yersinia pestis biovar Orientalis str. IP275	EamA	FecCD
Yersinia pestis bv. Antiqua (strain Antiqua; A0A0E1NTQ7)	EamA	FecCD
Yersinia pestis bv. Antiqua (strain Antiqua; A0A0H2Y4T7)	EamA	FecCD
Yersinia pestis bv. Antiqua (strain Nepal516)	EamA	FecCD
Yersinia pseudotuberculosis (A0A0T9J5L2)	EamA	FecCD
Yersinia pseudotuberculosis (A0A0T9LUS4)	EamA	FecCD
Yersinia pseudotuberculosis serotype I (strain IP32953)	EamA	FecCD
Yersinia pseudotuberculosis serotype O:1b (strain IP 31758)	EamA	FecCD

Yersinia pseudotuberculosis serotype O:3 (strain YPIII)	EamA	FecCD
Yersinia similis (A0A0T9P242)	EamA	FecCD
Yersinia wautersii (A0A0E8XDS6)	EamA	FecCD
Enterobacter cloacae (A0A157U2K9)	EamA	TonB
Pseudomonas aeruginosa (A0A077JXG2)	EamA	TonB
Pseudomonas aeruginosa (A0A0C7AQP6)	EamA	TonB
Pseudomonas aeruginosa (A0A0V3SFR0)	EamA	TonB
Pseudomonas aeruginosa (strain ATCC 15692 / DSM 22644 / CIP 104116 / JCM 14847 / LMG 12228 / 1C / PRS 101 / PAO1)	EamA	TonB
Pseudomonas aeruginosa (strain PA7)	EamA	TonB
Pseudomonas aeruginosa (strain UCBPP-PA14)	EamA	TonB
Pseudomonas aeruginosa BL04	EamA	TonB
Pseudomonas aeruginosa DK1	EamA	TonB
Pseudomonas aeruginosa MTB-1	EamA	TonB
Pseudomonas aeruginosa str. Stone 130	EamA	TonB
Pseudomonas aeruginosa VRFPA01	EamA	TonB
Pseudomonas aeruginosa VRFPA03 (no hemolysin)	EamA	TonB
Anaerobacillus macyae (A0A0J6D1P8)	EamA	ABC
Bacillus aminovorans (A0A177KXU3)	EamA	ABC
Bacillus marmarensis DSM 21297	EamA	ABC
Bacillus sp. FJAT-18019	EamA	ABC
Fictibacillus arsenicus (A0A1B1Z2E6)	EamA	ABC
Paenibacillus elgii (A0A165QP91)	EamA	ABC
Paenibacillus sp. FJAT-22460	EamA	ABC
Paenibacillus tyrfis (A0A081PAT8)	EamA	ABC
Fictibacillus phosphorivorans (A0A160IK29)	EamA	n/a
Fusobacterium mortiferum ATCC 9817 (C3WBZ5)	EamA	n/a
Actinokineospora spheciospongiae	MFS	None
Actinosynnema mirum (strain ATCC 29888 / DSM 43827 / NBRC 14064 / IMRU 3971)	MFS	None
Aneurinibacillus migulanus (Bacillus migulanus)	MFS	None
Bacillus halodurans (strain ATCC BAA-125 / DSM 18197 / FERM 7344 / JCM 9153 / C-125)	MFS	None
Bacillus okuhidensis	MFS	None
Glutamicibacter arilaitensis (strain DSM 16368 / CIP 108037 / IAM 15318 / JCM 13566 / Re117) (Arthrobacter arilaitensis)	MFS	None
Paenibacillus glucanolyticus	MFS	None
Paenibacillus mucilaginosus (strain KNP414)	MFS	None
Paenibacillus mucilaginosus 3016	MFS	None
Paenibacillus mucilaginosus K02	MFS	None
Paenibacillus naphthalenovorans	MFS	None
Paenibacillus sp. A3	MFS	None

Paenibacillus sp. FSL R5-192	MFS	None
Paenibacillus sp. FSL R5-808	MFS	None
Paenibacillus sp. FSL R7-0273	MFS	None
Paenibacillus sp. HGF5	MFS	None
Paenibacillus sp. TCA20	MFS	None
Paenibacillus sp. VT-400	MFS	None
Staphylococcus argenteus	MFS	None
Staphylococcus aureus	MFS	None
Staphylococcus aureus	MFS	None
Staphylococcus aureus	MFS	None
Staphylococcus aureus	MFS	None
Staphylococcus aureus	MFS	None
Staphylococcus aureus	MFS	None
Staphylococcus aureus	MFS	None
Staphylococcus aureus (strain COL)	MFS	None
Staphylococcus aureus (strain Mu50 / ATCC 700699)	MFS	None
Staphylococcus aureus (strain MW2)	MFS	None
Staphylococcus aureus (strain N315)	MFS	None
Staphylococcus aureus (strain NCTC 8325)	MFS	None
Staphylococcus aureus (strain Newman)	MFS	None
Staphylococcus aureus (strain USA300)	MFS	None
Staphylococcus aureus subsp. aureus	MFS	None
Staphylococcus aureus subsp. aureus	MFS	None
Staphylococcus aureus subsp. aureus 71193	MFS	None
Staphylococcus aureus subsp. aureus CN1	MFS	None
Staphylococcus aureus subsp. aureus MN8	MFS	None
Staphylococcus aureus subsp. aureus USA300_TCH959	MFS	None
Staphylococcus aureus subsp. aureus Z172	MFS	None
Staphylococcus capitis	MFS	None
Staphylococcus capitis	MFS	None
Staphylococcus caprae M23864:W1	MFS	None
Staphylococcus chromogenes MU 970	MFS	None
Staphylococcus epidermidis	MFS	None
Staphylococcus epidermidis	MFS	None
Staphylococcus epidermidis (strain ATCC 12228)	MFS	None
Staphylococcus epidermidis (strain ATCC 35984 / RP62A)	MFS	None
Staphylococcus epidermidis M23864:W2(grey)	MFS	None
Staphylococcus epidermidis W23144	MFS	None

Staphylococcus epidermidis WI09	MFS	None
Staphylococcus hyicus	MFS	None
Staphylococcus pasteuri	MFS	None
Staphylococcus pasteuri SP1	MFS	None
Staphylococcus pseudintermedius	MFS	None
Staphylococcus pseudintermedius	MFS	None
Staphylococcus pseudintermedius	MFS	None
Staphylococcus pseudintermedius (strain HKU10-03)	MFS	None
Staphylococcus schleiferi	MFS	None
Staphylococcus sp. DORA_6_22	MFS	None
Staphylococcus sp. HGB0015	MFS	None
Staphylococcus warneri (strain SG1)	MFS	None
Staphylococcus xylosus	MFS	None
Streptococcus pneumoniae	MFS	None
Streptococcus pneumoniae	MFS	None
Bacillus cereus HuB4-4	MFS	NAS
Bacillus gaemokensis	MFS	NAS
Bacillus hemicellulosilyticus JCM 9152	MFS	NAS
Bacillus sonorensis L12	MFS	NAS
Brevibacillus agri BAB-2500	MFS	NAS
Glutamicibacter arilaitensis	MFS	NAS
Paenibacillus amylolyticus	MFS	NAS
Paenibacillus sp. 1ZS3-15	MFS	NAS
Paenibacillus sp. FSL R7-269	MFS	NAS
Paenibacillus sp. HGF7	MFS	NAS
Paenibacillus sp. HGF7	MFS	NAS
Staphylococcus caprae M23864:W1	MFS	NAS
Bacillus cereus	MFS	Methyltrans
Bacillus litoralis	MFS	Methyltrans
Bacillus mycoides	MFS	Methyltrans
Bacillus sp. SA1-12	MFS	Methyltrans
Brevibacillus brevis (Bacillus brevis)	MFS	Methyltrans
Brevibacillus brevis (strain 47 / JCM 6285 / NBRC 100599)	MFS	Methyltrans
Brevibacillus parabrevis	MFS	Methyltrans
Brevibacillus sp. BC25	MFS	Methyltrans
Paenibacillus sp. A59	MFS	Methyltrans
Paenibacillus sp. AD87	MFS	Methyltrans
Paenibacillus sp. BIHB4019	MFS	Methyltrans

Paenibacillus sp. Leaf72	MFS	Methyltrans
Paenibacillus sp. Root52	MFS	Methyltrans
Staphylococcus agnetis	MFS	Methyltrans
Paenibacillus sp. HGF7 (F5LJV0)	MFS	n/a
Paenibacillus sp. MAEPY2 (A0A0A2UDG7)	MFS	n/a
Staphylococcus agnetis (A0A0S2F2G7)	MFS	n/a
Staphylococcus aureus (A0A0G2LVZ8)	MFS	n/a
Staphylococcus haemolyticus (A0A0U0VDE2)	MFS	n/a
