# Supplementary material for: The Metallophore Staphylopine Enables Staphylococcus aureus To Compete with the Host for Zinc and Overcome Nutritional Immunity
Source: mBio. 2017 Oct 31;8(5):e01281-17. doi: 10.1128/mBio.01281-17 (PMC5666155; doi:10.1128/mBio.01281-17)
Supplement: TABLE S2 [file mbo005173560st2.docx]

**Table S2. Primers used in this study.**

| Name | Sequence |
| --- | --- |
| *cntA* KO 5’ Fwd | GGGGACAAGTTTGTACAAAAAAGCAGGCTTTCTCAACTTATCTTGGCGATACACGTATTG |
| *cntA* KO 5’ Rev | ATTGCTCCTTTATTTATATTTTCTCATTTGCTTTTCCTCTTTCTAAATTG |
| *cntA* KO 3’ Fwd | GAAAAGCAAATGAGAAAATATAAATAAAGGAGCAATTAGATGTTCAAATTTATC |
| *cntA* KO 3’ Rev | GGGGACCACTTTGTACAAGAAAGCTGGGTTACAATAATGCCTAAAGCAATTACTGCACC |
| *cntKLM* KO 5’ Fwd | GGGGACAAGTTTGTACAAAAAAGCAGGCTGTCATAGTGAAAGATCGAGAGATTAACACG |
| *cntKLM* KO 5’ Rev | ATTATGAAAGCCGATTCATACGACACTCCTTTAGATG |
| *cntKLM* KO 3’ Fwd | TATGAATCGGCTTTCATAATAAGGGTTTGAAGTTTTATAATAGAAA |
| *cntKLM* KO 3’ Rev | GGGGACCACTTTGTACAAGAAAGCTGGGTCCAGAATTGACAACTAACATTTTCGTATTC |
| *adcA* comp Fwd | ACGTCATATGAAAAAGAAATTAGGTATGTTACTTCTTGTACC |
| *adcA* com Rev | AGCTGGATCCGAAATAATGCGCTAACATTTCTTCTTTGATTG |
| *cntA* comp Fwd | AGCTGGTACCGCAACTTATCAATTTAGAAAGAGGAAAAGC |
| *cntA* comp Rev | CGATGAGCTCGCTCCTTTATTTATACTGCATTTCATTGAATGG |
| *cntKLM* comp Fwd | AGCTGGTACCCAGAGGCTCTAAAAATACATCTAAAGGAGTG |
| *cntKLM* comp Rev | CGATGAGCTCCCCTTATTATGAAAGCGTTCTATTGATTTCC |
| p*adcA* Fwd | GTCACTGCAGCCATTATTAAATCTAAAAGTCACACTATAAAAATAGG |
| p*adcA* Rev | CGTAGGTACCCATAGTCACCCTCCTAAATAGTAATCTTTACG |
| p*cnt* Fwd | GTCACTGCAGGCTTTTTTGGTGCGTATTGTATGGGC |
| p*cnt* Rev | ACTGGGTACCACGACACTCCTTTAGATGTATTTTTAGAGCCTC |
