# Supplementary material for: The Metallophore Staphylopine Enables Staphylococcus aureus To Compete with the Host for Zinc and Overcome Nutritional Immunity
Source: mBio. 2017 Oct 31;8(5):e01281-17. doi: 10.1128/mBio.01281-17 (PMC5666155; doi:10.1128/mBio.01281-17)
Supplement: TABLE S3 [file mbo005173560st3.docx]

**Table S3. Strains used in this study.**

| Bacterial Strains | Genotype | Source | Identifier |
| --- | --- | --- | --- |
| *Staphylococcus aureus* Newman | Wild type | This study | N/A |
| Newman Δ*adcA* | *adcA::erm* | This study | N/A |
| Newman Δ*cntA* | Δ*cntA* | This study | N/A |
| Newman Δ*adcA*Δ*cntA* | *adcA::erm*Δ*cntA* | This study | N/A |
| Newman Δ*cntKLM* | Δ*cntKLM* | This study | N/A |
| Newman Δ*adcA*Δ*cntKLM* | *adcA::erm*Δ*cntKLM* | This study | N/A |
| Newman pRMC2 | Wild type carrying pRMC2 | This study | N/A |
| Newman Δ*cntA* pRMC2 | Δ*cntA* carrying pRMC2 | This study | N/A |
| Newman Δ*cntA* pRMC2::*cntA* | Δ*cntA* carrying pRMC2::*cntA* | This study | N/A |
| Newman Δ*adcA*Δ*cntA* pRMC2 | *adcA::erm*Δ*cntA* carrying pRMC2 | This study | N/A |
| Newman Δ*adcA*Δ*cntA* pRMC2::*cntA* | *adcA::erm*Δ*cntA* carrying pRMC2::*cntA* | This study | N/A |
| Newman Δ*adcA*Δ*cntA* pOS1plgt::*adcA* | *adcA::erm*Δ*cntA* carrying pOS1plgt::*adcA* | This study | N/A |
| Newman Δ*cntKLM* pRMC2 | Δ*cntKLM* carrying pRMC2 | This study | N/A |
| Newman Δ*cntKLM* pRMC2::*cntKLM* | Δ*cntKLM* carrying pRMC2::*cntKLM* | This study | N/A |
| Newman Δ*adcA*Δ*cntKLM* pRMC2 | *adcA::erm*Δ*cntKLM* carrying pRMC2 | This study | N/A |
| Newman Δ*adcA*Δ*cntKLM* pRMC2::*cntKLM* | *adcA::erm*Δ*cntKLM* carrying pRMC2::*cntKLM* | This study | N/A |
| Newman Δ*adcA*Δ*cntKLM* pOS1plgt | *adcA::erm*Δ*cntKLM* carrying pOS1plgt | This study | N/A |
| Newman Δ*adcA*Δ*cntKLM* pOS1plgt::*adcA* | *adcA::erm*Δ*cntKLM* carrying pOS1plgt::*adcA* | This study | N/A |
| Newman pEmpty | Wild type carrying pAH5::empty | (30) | N/A |
| Newman pAH5::*padcA* | Wild type carrying pAH5::*padcA* | This study | N/A |
| Newman pAH5::*pcnt* | Wild type carrying pAH5::*pcnt* | This study | N/A |
| Newman Δ*zur* pEmpty | Δ*zur* carrying pAH5::empty | This study | N/A |
| Newman Δ*zur* pAH5::*padcA* | Δ*zur* carrying pAH5::*padcA* | This study | N/A |
| Newman Δ*zur* pAH5::*pcnt* | Δ*zur* carrying pAH5::*pcnt* | This study | N/A |
| Newman Δ*fur* pEmpty | *fur::tet* carrying pAH5::empty | This study | N/A |
| Newman Δ*fur* pAH5::*padcA* | *fur::tet* carrying pAH5::*padcA* | This study | N/A |
| Newman Δ*fur* pAH5::*pcnt* | *fur::tet* carrying pAH5::*pcnt* | This study | N/A |
| Newman Δ*mntR* pEmpty | Δ*mntR* carrying pAH5::empty | This study | N/A |
| Newman Δ*mntR* pAH5::*padcA* | Δ*mntR* carrying pAH5::*padcA* | This study | N/A |
| Newman Δ*mntR* pAH5::*pcnt* | Δ*mntR* carrying pAH5::*pcnt* | This study | N/A |
| *Staphylococcus aureus* USA300 (JE2) | Wild type | Nebraska Transposon Mutant Library | NR-46543 |
| USA300 (JE2) Δ*adcA* | *adcA::erm* | Nebraska Transposon Mutant Library | NR-47111 |
| USA300 (JE2) Δ*cntA* | *cntA::erm* | Nebraska Transposon Mutant Library | NR-47717 |
| USA300 (JE2) Δ*cntB* | *cntB::erm* | Nebraska Transposon Mutant Library | NR-47752 |
| USA300 (JE2) Δ*cntC* | *cntC::erm* | Nebraska Transposon Mutant Library | NR-47427 |
| USA300 (JE2) Δ*cntD* | *cntD::erm* | Nebraska Transposon Mutant Library | NR-47095 |
| USA300 (JE2) Δ*cntF* | *cntF::erm* | Nebraska Transposon Mutant Library | NR-48146 |
| USA300 (JE2) Δ*cntK* | *cntK::erm* | Nebraska Transposon Mutant Library | NR-47202 |
| USA300 (JE2) Δ*cntL* | *cntL::erm* | Nebraska Transposon Mutant Library | NR-48167 |
| USA300 (JE2) Δ*cntM* | *cntM*::erm | Nebraska Transposon Mutant Library | NR-47529 |
