# Supplementary material for: The Metallophore Staphylopine Enables Staphylococcus aureus To Compete with the Host for Zinc and Overcome Nutritional Immunity
Source: mBio. 2017 Oct 31;8(5):e01281-17. doi: 10.1128/mBio.01281-17 (PMC5666155; doi:10.1128/mBio.01281-17)
Supplement: TABLE S4 [file mbo005173560st4.docx]

**Table S4. Plasmids used in this study.**

| Name | Description | Source |
| --- | --- | --- |
| pOS1plgt | complementation vector containing the lgt promoter |  |
| pAdcA | pOS1plgt::*adcA* | This study |
| pRMC2 | Anhydrotetracycline inducible vector | (72) |
| pCntA | pRMC2::*cntA* | This study |
| pCntKLM | pRMC2::*cntKLM* | This study |
| pAH5 | YFP reporter plasmid | (30) |
| pEmpty | pAH5 containing YFP lacking a promoter | (73) |
| P_adcA_-YFP | pAH5::*padcA* | This study |
| P_cnt_-YFP | pAH5::*pcnt* | This study |
| pS100A9 | CP expression construct | (20) |
| pS100A8 | CP expression construct | (20) |
| pS100A9 H91N, H95N | CP ∆Mn/Zn site expression construct | (20) |
| pS100A8 H17N, H27N | CP ∆Mn/Zn site expression construct | (20) |
| pS100A9 D30S, H20N | CP ∆Zn site expression construct | (20) |
| pS100A8 H83N, H87N | CP ∆Zn site expression construct | (20) |
